# Evaluating Depression Among Acne Vulgaris Patients Treated With Isotretinoin

**DOI:** 10.7759/cureus.12126

**Published:** 2020-12-17

**Authors:** Bader N Algamdi, Hussam W ALdahlan, Hussain ALALhareth, Radhwan Alghamdi, Moath T Alkhouzaie, Nawaf ALahmari, Abdulrahman Alhudaifi, Yaser Alkasih, Wyle Elyahia, Khalid ALghamdi

**Affiliations:** 1 Dermatology, King Fahd Hospital of the University, Khobar, SAU; 2 Vascular surgery, King Fahad University Hospital, Khobar, SAU

**Keywords:** acne vulgaris, isotretinoin, depression

## Abstract

Background

Isotretinoin is the most effective treatment for moderate to severe acne. However, isotretinoin has many side effects related to its use. Since 1983, when Hetzen reported the first occurrence of new depressive symptoms in patients treated with isotretinoin, a lot of controversies emerged regarding the causal relationship between isotretinoin and depression.

Objective

To evaluate depression among acne patients treated with isotretinoin versus doxycycline at King Fahad Hospital of the University between December 2019-March 2020.

Methods

Using the Global Acne Grading System, patients aged 18 - 30 years old with moderate to severe acne vulgaris who have not received isotretinoin previously and has no personal or family history of any psychiatric illnesses, were evaluated for depression using the patient health questionnaire-9 before starting treatment and 8 weeks after. Twenty-nine patients had met the inclusion criteria and were included in the study.

Results

Of the 29 patients included, 18 patients completed the study (nine males, nine females). Twelve patients received isotretinoin 0.5mg/kg (study group) and six patients received doxycycline 100mg (control group). The mean depression score for the isotretinoin group has decreased from (4 ± 2.48) to (3.08 ± 2.84) but the result was statistically insignificant with a p-value of 0.19, CI (-5.28, 2.36). For the doxycycline group, the mean depression score has decreased from (5.5 ± 2.5) to (2.83 ± 0.75) with a p-value of 0.043, CI (0.12, 5.21). There was no statistically significant difference in the mean depression score between the two groups after 8 weeks of starting treatment [p-valve 0.837, CI (-2.28, 2.78)].

Conclusion

This study showed that, after 8 weeks of starting treatment, isotretinoin at 0.5 mg/kg has no risk of developing depression. The results of this study did not reveal a direct relationship between the use of isotretinoin and the development of depression. Furthermore, optimum control and treatment of acne vulgaris have shown to improve depression scores.

## Introduction

Acne Vulgaris is a disease of the pilosebaceous units of skin, affecting around 85% of adolescents and young adults globally [1,2]. It is characterized by the development of multiple comedones, papules, pustules, and nodules, which can be occasionally complicated by scarring. These lesions appear on the face, the upper trunk, or the proximal upper extremities and can lead to psychological disturbances in some patients [3-5].

Isotretinoin is the most effective treatment for moderate to severe acne. However, isotretinoin has many side effects related to its use. Since 1983 when Hetzen reported the first occurrence of new depressive symptoms in patients treated with isotretinoin, a lot of controversies emerged regarding the causal relationship between isotretinoin and depression [4]. In their study, Schaffer et al. confirms that patients with a history of bipolar disease are at higher risk of developing depression induced by isotretinoin [6]. However, a systematic review and meta-analysis done by Huang and Cheng concluded that no association between using isotretinoin and increased risk of depression exists, moreover symptoms of depression had improved by isotretinoin [7]. To date, no evidence-based study confirms a positive relationship between depression and isotretinoin [8].

The aim of this study is to evaluate depression among patients with moderate to severe acne vulgaris who are treated with isotretinoin compared with doxycycline by conducting a questionnaire-based prospective cohort study at King Fahad Hospital of the university in Khobar, Saudi Arabia.

## Materials and methods

Subjects

This study has been approved by Imam Abdulrahman Bin Faisal University IRB (IRB -UGS-2019-01-372) in December 2019 and was conducted at the dermatology outpatient clinic in King Fahd Hospital of the University, Khobar, Saudi Arabia, from November 2019 to March 2020. All participants have signed informed consent of voluntary participation and have informed that this study will not affect their treatment plan and that they have the right to withdraw from the study at any time with no consequences. Male and female patients aged 18 to 30 years old who were diagnosed with moderate to severe acne vulgaris were included in the study. A personal or family history of psychiatric illness, a history of using antipsychotic or antidepressant drugs, and a history of receiving a previous course of isotretinoin or using recreational drugs were considered as exclusion criteria.

Study design

This is a cohort prospective, questionnaire-based study evaluating depressive symptoms among two groups of patients with moderate to severe acne vulgaris. The first group included patients who will be started on isotretinoin 0.5 mg/kg (study group), while the second group included those who will receive 100 mg doxycycline (control group). Patients were not randomized to these groups as their treatment plan was based on the severity of the lesions, previous treatments, general health, and patient preference. Both groups were evaluated for depressive symptoms at baseline before starting the treatment, and then after 8 weeks.

Materials

Acne severity was evaluated using the Global Acne Grading System (GAGS). A total score of 18 and lower was considered mild acne, 19-30 moderate acne, 31-38 severe acne, and 39 or more was considered very severe [9]. Depressive symptoms were self-reported by the patients using the Patient Health Questionnaire 9 (PHQ-9) and a cut-off point of 10 (as recommended) was used [10].

Procedure

All patients who met the inclusion criteria were offered to voluntarily participate in the study. Those who agreed were asked to sign an informed consent before their enrollment in the study. Each patient was given a code number to ensure the confidentiality of their information and was asked to fill the PHQ-9 by him/her self. After 8 weeks of starting the treatment, they were asked to fill the PHQ-9 again. 

Data analysis

SPSS version 25 was used. The depression score for each group was analyzed by the paired t-test. The difference between isotretinoin and doxycycline on depression scores after 8 weeks of treatment was analyzed by the independent student`s t-test. A p-value < 0.05 was considered significant.

## Results

A total of 29 patients had met the inclusion criteria and were included in the study. Eleven patients were later excluded because they lost to follow up (Table 1).

**Table 1:** Demographic characteristics for all patients

	N	Gender		Mean age	Acne severity		Mean
All patients included	29	17 Males (58%)	12 Females (42%)	21.62 ± 2.9 years old	22 Moderate (75%)	7 severe (25%)	Mean 26.48 ± 5.3
Patients completed study	18	9 Males (50%)	9 Females (50%)	21.7 ± 2.7 years old	13 Moderate (72%)	5 severe (28%)	Mean 26.5 ± 5.2

The remaining 18 patients who completed the study were nine males (50%) and nine females (50%) with a mean age of (21.7 ± 2.7). 13 patients (72%) had moderate acne severity while five patients (28%) had severe acne, and the mean acne severity score was 26.5 ± 5.2 (Table 2).

**Table 2:** Demographic characteristics for both groups.

	N	Gender		Mean age	Acne severity	
Isotretinoin	12	5 Males (42%)	7 Females (58%)	21.6 ± 2.5 years old	9 Moderate (75%)	3 severe (25%)
Doxycycline	6	4 Males (67%)	2 Females (33%)	21.8 ± 3.6 years old	4 Moderate (67%)	2 severe (33%)

Twelve patients were started on 0.5 mg/kg of isotretinoin (67%) and six patients were on 100 mg doxycycline (33%). At baseline, the mean depression score for patients on isotretinoin was (4.00 ± 2.48) and was (5.5 ± 2.50) for the doxycycline group. In both groups, none of the patients had a depression score of 10 or more. After 8 weeks of follow-up, patients on isotretinoin and doxycycline had a mean depression score of (3.08 ± 2.84) and (2.83 ± 075) respectively (Table 3).

**Table 3:** The depression score of the PHQ-9 scale for both groups

	Drug used	N	Mean	SD
At base line	Isotretinoin	12	4	2.48
	Doxycycline	6	5.5	2.51
After 8 weeks of starting treatment	Isotretinoin	12	3.0833	2.84
	Doxycycline	6	2.8333	0.75

We found that the mean depression score had decreased in both the isotretinoin and the doxycycline groups with a p-value of (0.190) and (0.043) respectively (Table 4). When comparing the two groups there was no statistically significant difference between isotretinoin and doxycycline after 8 weeks of treatment with a p-value of 0.837 (Table 5).

**Table 4:** Paired t-test for each group comparing between baseline and 8 weeks of starting treatment

	mean	SD	t	df	p	CI
Isotretinoin	0.92	2.27	1.396	11	0.19	-5.28,2.36
Doxycycline	2.67	2.42	2.697	5	0.043	0.12,5.21

**Table 5:** Independent Student`s t-test for comparing isotretinoin with doxycycline after 8 weeks of starting treatment.

	t	df	p	CI
After 8 weeks	0.209	16	0.837	-2.28,2.78

## Discussion

In our study, no increase in depression score was observed among the two groups. Rather, there was a decrease in the score, although it was statistically insignificant for the isotretinoin group. The results of this study did not reveal a direct relationship between the use of isotretinoin and the development of depression. These findings are in agreement with other studies [7,11,12]

A metanalysis done by Huang et al. illustrated that isotretinoin therapy for acne at the recommended doses (0.5-1 mg/kg) was not associated with an increased risk of depression. Moreover, symptoms of depression showed marked improvement in most of the patients [7]. Another metanalysis done by Li et al. concluded that the use of isotretinoin in acne patients might be associated with improvement of depressive symptoms [11].

A prospective study by Gnanaraj et al. concluded that the depression score was higher before the initiation of isotretinoin therapy. However, during therapy, the depression score was shown to decrease steadily and the reduction at the end of three months of therapy was statistically significant. Furthermore, during the 6 months follow-up period, there was no worsening of depression score nor the development of suicidal thoughts in any patient [12].

The effect of isotretinoin on depression remains unclear. Theoreticality, isotretinoin is a fat-soluble compound and can cross the blood-brain barrier causing changes through the retinoid receptors [13]. This was emphasized by Schaffer et al. in their study where they found that patients with a history of bipolar disease are at higher risk of developing depression induced by isotretinoin [6]. Likewise, acne itself can lead to an increased risk of psychological disturbance due to its apparent disfigurement [5,11]. Thus, the appropriate treatment of acne may have contributed to a lower depression score observed in this study.

To prove the exact relation between isotretinoin and depression a placebo-controlled prospective study is needed [5].

## Conclusions

This study showed that, after 8 weeks of starting treatment, isotretinoin at 0.5 mg/kg has no risk of developing depression. The results of this study did not reveal a direct relationship between the use of isotretinoin and the development of depression. Furthermore, optimum treatment of acne vulgaris has shown improvement of depression score. Additionally, patients on isotretinoin should be educated regarding the possible symptoms of depression and the need to report it.

No conclusion can be made regarding the use of isotretinoin in patients with a history of psychiatric illness. However, if isotretinoin was needed in such patients, we recommend a multidisciplinary approach by involving a psychiatric physician to evaluate and regularly follow up with these patients.

## References

[1] Aydemir EH (2014). Acne vulgaris. Turk Pediatr Ars.

[2] White GM (1998). Recent findings in the epidemiologic evidence, classification, and subtypes of acne vulgaris. In: Journal of the American Academy of. Dermatology.

[3] Jick SS, Kremers HM, Vasilakis-Scaramozza C (2000). Isotretinion use and risk of depression, psychotic symptoms, suicide, and attempted suicide. Arch Dermatol.

[4] Hetzen PG, Carney JF, Walker AE, Stewart JJ (1983). Depression—a side effect of 13-cis-retinoic acid therapy. J Am Acad Dermatol.

[5] Misery L (2011). Consequences of psychological distress in adolescents with acne. J Invest Dermatol.

[6] Schaffer LC, Schaffer CB, Hunter S, Miller A (2010). Psychiatric reactions to isotretinoin in patients with bipolar disorder. J Affect Disord.

[7] Huang YC, Cheng YC (2017). Isotretinoin treatment for acne and risk of depression: a systematic review and meta-analysis. J Am Acad Dermatol.

[8] Rea S, Tucker S, Frittelli V, Gunnarsson R (2018). A feasibility study for a triple-blind randomized controlled trial investigating the effects of oral isotretinoin on mood and quality of life in patients with acne vulgaris. Clin Exp Dermatol.

[9] Doshi A, Zaheer A, Stiller MJ (1997). A comparison of current acne grading systems and proposal of a novel system. Int J Dermatol.

[10] Manea L, Gilbody S, McMillan D (2012). Optimal cut-off score for diagnosing depression with the Patient Health Questionnaire (PHQ-9): a meta analysis. CMAJ.

[11] Li C, Chen J, Wang W, Ai M, Zhang Q, Kuang L (2019). Use of isotretinoin and risk of depression in patients with acne: a systematic review and meta-analysis. BMJ Open.

[12] Gnanaraj P, Karthikeyan S, Narasimhan M, Rajagopalan V (2015). Decrease in “Hamilton rating scale for depression” following isotretinoin therapy in acne: an open-label prospective study. Indian J Dermatol.

[13] Diehl DJ, Gershon S (1992). The role of dopamine in mood disorders. Compr Psychiatry.

